# An Integrated Account of Expert Perspectives on Functioning in Schizophrenia

**DOI:** 10.3390/jcm10184223

**Published:** 2021-09-17

**Authors:** Laura Nuño, Georgina Guilera, Emilio Rojo, Juana Gómez-Benito, Maite Barrios

**Affiliations:** 1 Clinical Institute of Neuroscience (ICN), Hospital Clinic, 08036 Barcelona, Spain; 2 Department of Social Psychology and Quantitative Psychology, University of Barcelona, 08007 Barcelona, Spain; gguilera@ub.edu (G.G.); juanagomez@ub.edu (J.G.-B.); mbarrios@ub.edu (M.B.); 3 Group on Measurement Invariance and Analysis of Change (GEIMAC), Institute of Neurosciences, University of Barcelona, 08007 Barcelona, Spain; 4 Hospital Benito Menni CASM, Sisters Hospitallers, 08830 Sant Boi de Llobregat, Spain; erojo.hbmenni@hopitalarias.es; 5 Department of Psychiatry, International University of Catalonia, 08007 Barcelona, Spain

**Keywords:** schizophrenia, rehabilitation, mental disorders, public mental health, Delphi studies

## Abstract

An integrated and interdisciplinary care system for individuals with schizophrenia is essential, which implies the need for a tool that assesses the difficulties and contextual factors of relevance to their functioning, and facilitates coordinated working across the different professions involved in their care. The International Classification of Functioning, Disability and Health Core Sets (ICF-CS) cover these requirements. This study aimed to evaluate the content validity of the ICF-CSs for schizophrenia from the perspective of experts. Six three-round Delphi studies were conducted with expert panels from different professional backgrounds which have played a significant role in the treatment of individuals with schizophrenia (psychiatry, psychology, nursing, occupational therapy, social work and physiotherapy). In total, 790 experts from 85 different countries participated in the first round. In total, 90 ICF categories and 28 Personal factors reached expert consensus (reached consensus from four or more professional perspectives). All the categories in the brief version of the ICF-CS for schizophrenia reached consensus from all the professional perspectives considered. As for the comprehensive version, 89.7% of its categories reached expert consensus. The results support the worldwide content validity of the ICF-CSs for schizophrenia from an expert perspective and underline the importance of assessing functioning by considering all the components implied.

## 1. Introduction

Schizophrenia has long been considered a chronic mental illness that is predestined to irreversible progressive deterioration [1]. However, the scientific evidence has shown that the course and evolution of the illness are very heterogeneous [2,3], from cases that require several hospitalizations and have an important detriment of their functioning to cases that only show one episode, followed by symptom remission and functional recovery. A recent meta-analysis [4] showed that individuals with schizophrenia achieve symptom remission in 56% of cases, while recovery, which includes satisfactory psychosocial functioning, is achieved in around 30% of cases. Therefore, personal, social and occupational functioning is usually impaired in this population [5,6], but recovery is possible and should be a priority in the treatment of persons diagnosed with schizophrenia.

In view of this, an integrated and interdisciplinary care system is needed [7] where professionals from different fields who treat people diagnosed with this health condition work together not only to address their symptoms, but also the difficulties they present in their daily functioning, their personal characteristics and the environmental factors that affect them.

In the case of individuals diagnosed with schizophrenia, the literature has shown that the inclusion of psychiatrists [2,4], psychologists [8,9], nurses [10,11], occupational therapists [12,13], social workers [14,15] and physiotherapists [16,17] in interdisciplinary mental health teams providing integrative care to this population has substantial effects improving clinical, social and assistance outcomes [18,19,20].

**Figure 1:**
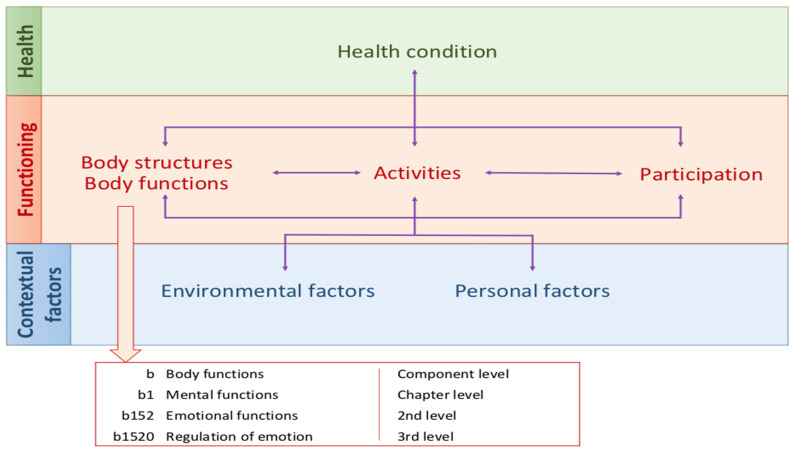
The ICF structure.

This shift in the therapeutic approach highlights the need for a tool that can assess the full spectrum of difficulties in functioning that a person may have, and all the contextual variables involved and which facilitates the coordination and joint work among all the professions involved in the recovery process. Moreover, achieving integrated care goals requires a common language and an understanding of the patient’s functioning problems among interdisciplinary team members. The International Classification of Functioning, Disability and Health (ICF [21]) covers all these requirements. The ICF, and the integrated biopsychosocial model on which it is based, represent a comprehensive and universally accepted framework for describing functioning, disability and health in persons with all types of health conditions. The ICF considers that problems associated with a disease can be related to *Body functions*, *Body structures*, and *Activities and Participation* in community life, which in turn are influenced by *Environmental factors* and *Personal factors.* Each of these components is structured hierarchically in chapters and categories (see Figure 1).

Given that it has more than 1400 categories, ICF Core Sets (ICF-CSs) linked to certain health conditions have been developed. The ICF-CSs consist of a list of the most relevant ICF categories for the description of the functioning and disability of persons living with a given health condition. In the case of schizophrenia, two versions of ICF-CSs have been developed following the methodology endorsed by the World Health Organization [22]: the brief and the comprehensive [23]. The Comprehensive ICF-CS for schizophrenia includes 97 categories covering the typical spectrum of problems in the functioning of patients with schizophrenia. The Brief ICF-CS for schizophrenia is a selection of 25 of these 97 categories, those considered the most important for the assessment and treatment of people with schizophrenia. Both ICF-CSs can be consulted at https://www.icf-research-branch.org/icf-core-sets-projects2/mental-health/icf-core-set-for-schizophrenia, accessed on 1 July 2021.

In order to apply the ICF-CSs in clinical practice, they must be validated through various sources of evidence. Hence, the goal of the present study is to evaluate the content validity of the ICF-CSs for schizophrenia from the perspective of experts in the treatment of this population, and to identify, from an expert perspective, the potential repercussions of this health condition in the functioning of persons diagnosed with this disorder. It is hypothesized that the problems, resources, and environmental factors that are represented in the ICF-CS for schizophrenia will coincide with those considered to be the most relevant for understanding and assessing the functioning of this population from the health professional perspective.

## 2. Materials and Methods

Six three-round Delphi studies were conducted with expert panels from different professional backgrounds, which have played a significant role in the treatment of persons with schizophrenia (i.e., psychiatry, psychology, nursing, occupational therapy, social work and physiotherapy). The purpose of the Delphi technique is to reach the consensus of a group of people possessing knowledge of a subject of interest (hereinafter referred to as “experts”). This is a multi-level procedure in which a series of rounds are conducted to gather information on a particular topic, so that each stage is built on the results of the previous stage, providing anonymous feedback to each participant on the opinion of the rest of the panel [24,25]. This methodology makes it easy to get the opinion of numerous experts from different backgrounds, moving from individual opinions into group consensus [26].

The Institutional Review Board Committee of the University of Barcelona approved the Study IBR00003099.

### 2.1. Participants

We aimed to obtain a sample of experts that reflected the worldwide variability of different variables considered to be interest: gender, age, years of experience and demographic region of origin. To this end, experts from around the world were recruited from a variety of sources, including through international associations of the analyzed professions, universities with internship programs for health professionals, and hospitals. Potential participants were also searched through a variety of bibliographical searches, LinkedIn contacts, and personal recommendations of the contacted experts. All of them were sent an initial invitation letter stating the criteria for participating in the study (i.e., being professionals in the specific profession of each Delphi study with at least one years’ experience in treating people diagnosed with schizophrenia). Specific knowledge of ICF was not required, as the responses had to be based on clinical experience. They were informed through a detailed description of the goals and the Delphi process, and they were asked for their socio-demographic and professional information.

In total, 1555 healthcare professionals agreed to participate, and from this set, those who fulfilled the inclusion criteria were invited to participate in the study (specifically, 443 psychiatrists, 223 psychologists, 160 nurses, 127 occupational therapists, 135 social workers and 22 physiotherapists).

In the first round of Delphi studies, 790 experts participated (71.2% of whom were invited to participate in the first round) from 85 different countries covering the six WHO regions. Table 1 shows the details of their socio-demographic and professional characteristics. A total of 638 participants (303 psychiatrists, 137 psychologists, 79 nurses, 73 occupational therapists, 36 social workers and 10 physiotherapists) completed the third round (80.8% compared to the first round).

### 2.2. Procedure

The six Delphi studies were conducted between 2016 and 2018. All studies followed the same design to ensure a high level of comparability between the results of each, and it has been detailed in the previous studies which describe the results obtained from each perspective: psychiatry [27], psychology [28], nursing [29], occupational therapy [30], social work [31] and physiotherapy [32]. Anonymity was guaranteed as the process was coordinated by a research team using an online platform or email, thus avoiding any interaction between the participants. The identity of the experts was not revealed to anyone but the research team and the feedback given was anonymous (the percentage of yes/no answers considering the expert panel as a whole), in order to ensure the independence of the participants’ opinions.

Each of the studies lasted two months, from the beginning of the first round to the end of the third round. The participants had two weeks to respond to each round. All the material and questionnaires were presented in five different languages (namely Chinese, English, French, Russian, and Spanish) in order to minimize potential language barriers and encourage maximum participation in different world regions. The answers for each Delphi round were collected via an online survey system (www.qualtrics.com, accessed on 1 July 2021).

**Figure 2:**
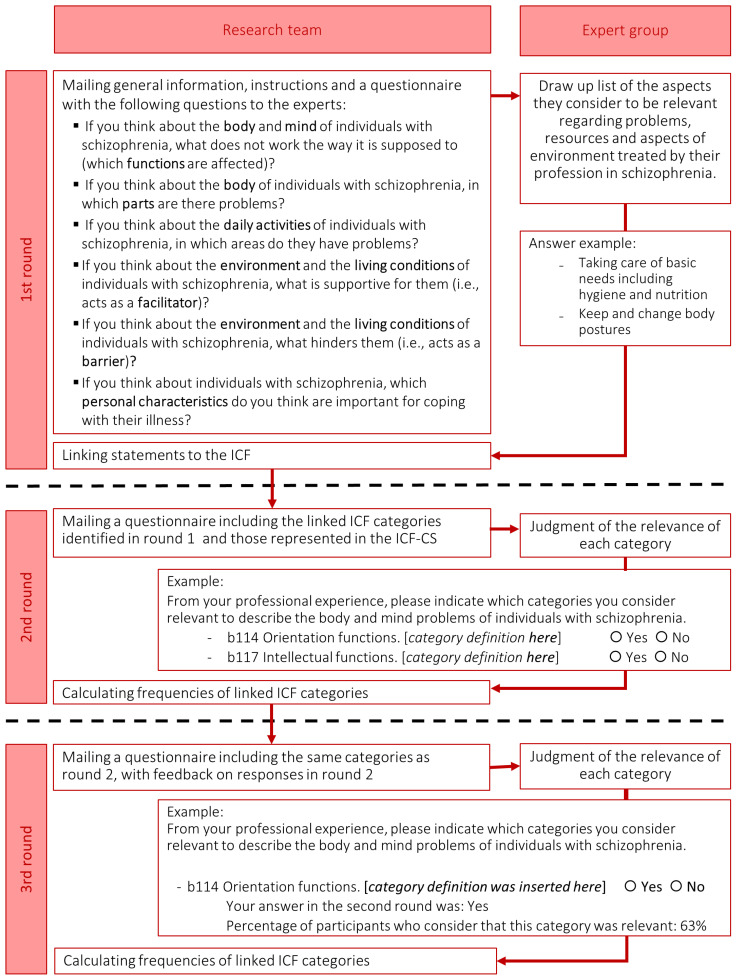
General procedure followed in each Delphi study.

For the first round, each participant was sent an email with a link to the survey webpage, asking them to list all aspects they considered relevant when assessing and/or treating people diagnosed with schizophrenia. To facilitate this, they were asked six open-ended questions covering all the ICF-CS components. Responses were not limited in terms of word length, although respondents were instructed to be brief and concise, and to avoid using abbreviations and vague technical terms. The general procedure followed in each Delphi study and the verbatim questions asked in the first round can be consulted in Figure 2.

Responses to the first round were linked to ICF categories by two health professionals following standardized rules [33,34]. Any disagreement between the two independent coders was reviewed and discussed by two other health professionals in order to reach a consensus. As *Personal factors* are not yet categorized in the current ICF system, the proposed categorization of Nuño et al. [27] was followed. Those ICF categories and *Personal factors* reported by at least 5% of the participants were selected for inclusion in the second and third Delphi rounds. If any category of ICF-CSs for schizophrenia was not included in this list, it was added.

In the second round, all the panelists who had responded in the first round were sent a list of the selected ICF categories, as well as a list of the categories proposed for *Personal factors*, together with their respective definitions. For example, for the category *b140 Attention functions*, the ICF definition (i.e., specific mental functions of focusing on an external stimulus or internal experience for the required period of time) and inclusions of the category (i.e., functions of sustaining attention, shifting attention, dividing attention, sharing attention; concentration; distractibility) were detailed. Participants were asked to judge, for each category, whether or not they considered the category to be relevant from their professional perspective for the evaluation and/or treatment of persons with schizophrenia. They were reminded that the aim was to obtain a final list short enough to be applicable in clinical practice and sufficiently comprehensive to cover the most important needs of people with schizophrenia.

Finally, in the third round, participants were asked to re-evaluate the same list of categories, this time taking into account the feedback they had received on the responses from the expert group as a whole and their own in the previous round.

### 2.3. Data Analysis

A descriptive analysis of the socio-demographic characteristics of the participants was conducted. Kappa coefficients and 95% bootstrapped confidence intervals (95% CI) were calculated to evaluate inter-coder reliability in the linking process of participants’ responses. For each Delphi study, the percentage of participants who selected each category as relevant in the second and third rounds was calculated. In the absence of a universally accepted definition of consensus [35], and given the experience of previous studies [36], the consensus was defined as an agreement among at least 75% of participants in the third round.

When performing the joint analysis of results, all categories that had reached consensus from the perspective of at least one profession were considered. Expert consensus was defined as an agreement among more than half of the professional perspectives considered (i.e., consensus by four or more expert perspectives for to that category).

All variable data were coded in Excel (2016) and analyzed using the Statistical Package for Social Sciences (SPSS, version 24) [37].

## 3. Results

### 3.1. Selected Categories from the Experts’ Perspective

In the first round, 20,551 concepts were extracted from the responses of the experts, which were linked to ICF categories and *Personal factors*. As a result of this process, different sets of categories were presented to each professional group according to the categories they had identified in the first round. Specifically, in the second and third rounds, between 110 and 135 ICF categories and between 24 and 35 *Personal factors* were presented to each expert group. Of these, 90 ICF categories and 28 *Personal factors* achieved expert consensus (reached consensus from the perspective of four or more professions).

### 3.2. Correspondence between Categories Which Reached Expert Consensus and the ICF-CSs for Schizophrenia

All the categories in the brief version of the ICF-CS for schizophrenia achieved consensus from all professional perspectives considered. Therefore, we will mainly focus on the comprehensive version of the ICF-CS for schizophrenia. All categories of the ICF-CS achieved consensus from the perspective of at least one professional group, and 89.7% (87 categories) achieved expert consensus.

More detailed information on the categories that achieved expert consensus and their correspondence with the categories present in the ICF-CS for schizophrenia can be found in Table 2. Moreover, the discrepancies between the results and the ICF-CS for schizophrenia are shown in Table 3, which details the specific categories that did not match between the set of categories that achieved expert consensus and the whole categories present in the ICF-CS for schizophrenia.

### 3.3. Personal Factors

Of all the *Personal factors* identified in the Delphi studies, 28 reached expert consensus, and 12 of them achieved consensus from all perspectives. The categories proposed as *Personal factors* that reached expert consensus, with information on from which professional perspectives they were considered and whether they reached consensus on each of them, can be consulted in S2.

## 4. Discussion

Through these studies, we identified the problems, resources, and environmental factors that health professionals most frequently encounter when treating people with schizophrenia. All the categories that form part of the ICF-CSs for schizophrenia achieved agreement from the perspective of at least one profession, and 89.7% of the categories of the comprehensive version achieved expert consensus. Moreover, 100% of the categories that make up the brief version of the ICF-CS achieved consensus from all of the perspectives considered. All this supports the high relevance of the categories that form part of the brief version. The following discussion will therefore focus on the joint analysis of the expert perspective in comparison with the comprehensive version of the ICF-CS for schizophrenia.

### 4.1. Body Functions

With regard to the *Body functions* component, 17 categories achieved expert consensus, coinciding with 16 of the 17 categories from this component represented in the ICF-CS for schizophrenia. Twelve of them achieved consensus from the perspective of all the expert groups considered, and all these categories belong to chapter *b1 Mental functions*, highlighting the relevance of this chapter for defining functioning in people with schizophrenia from the perspective of all health professions. All professionals agreed—with agreement from each perspective being higher than 95%—that categories referring to classical symptoms in schizophrenia, such as delusions and hallucinations (e.g., *b156 Perceptual functions* and *b160 Thought functions*), negative symptoms (*b130 Energy and drive functions* and *b152 Emotional functions*), and other typical alterations such as cognitive deficits (*b140 Attention functions* and *b164 Higher-level cognitive functions*) and psychosocial functions (*b122 Global psychosocial functions*), are crucial to consider when evaluating and treating this population.

These results also highlight the need for an interdisciplinary approach to all these functions. Interventions from the different professional profiles have proved to be effective for improving these areas of functioning. For example, antipsychotic treatment is the first-choice treatment for reducing positive symptoms and achieving remission of 80% of symptoms after the first year of treatment [38], but cognitive behavioral therapy [39], interventions of occupational therapy [12] and nursing [40] or exercises promoted by physiotherapists [41] have also been shown to be effective in reducing such symptoms. In this regard, social workers may also contribute to alleviating psychotic symptoms by conducting appropriate assessments and referrals [42]. Cognitive function is mainly improved through cognitive remediation therapy [43,44], but it has also been proven that it can improve through medical interventions [45], occupational therapy [46] and physiotherapeutic interventions [16]. These are some examples of how interventions carried out from each professional perspective can produce effects in these affected areas. However, these positive effects are even higher when applied by means of an integrated care approach, in which professionals from the different areas work together in a systematic way with the same objective [19,20].

It is also worth noting that the category *b126 Temperament and personality functions* achieved consensus from all of the perspectives considered, yet it is not represented in the ICF-CS for schizophrenia. This high rate of agreement is consistent with many studies in the literature that support the fact that this area may be affected in this population [47,48,49], and, therefore, its addition to the ICF-CS for schizophrenia should be considered.

Only one category from the *Body functions* component represented in the ICF-CS did not achieve consensus from the majority of the perspectives considered. This category was *b530 Weight maintenance functions*, which only reached consensus from the perspective of psychiatrists, nurses and physiotherapists. This suggests that this category is relevant to the assessment of, and interventions for, persons with schizophrenia, but may not be the most common target of the interventions of certain professionals, such as psychologists, which focus primarily on mental rather than other body functions, or OTs, which focus mainly on helping people recover and participate in significant life roles. However, weight gain and obesity are very prevalent in this population, increase the risk of weight-related health problems, such as adult-onset diabetes mellitus and cardiovascular disorders, and are related to reduced adherence with pharmacological interventions and quality of life [50]. Professionals such as psychologists, occupational therapists and social workers could play a significant role in reducing its incidence by promoting healthy habits and lifestyles in individuals with schizophrenia. In fact, first choice interventions to decrease and manage weight gain in this population are psychoeducation, diet, and physical activity interventions [51]. Therefore, these results show the need to raise awareness in certain professions about the relevance of weight-related health problems and the importance of intervening in them.

### 4.2. Body Structures

The ICF-CS for schizophrenia does not include any category from the *Body structures* component. However, all the expert panels agreed that brain structure was altered in individuals with schizophrenia, with agreement higher than 90% from all the perspectives in the category *s110 Brain structure*. This is supported by the literature, which suggests that the brain is the principle altered structure in this illness [52] and that other dysfunctions, such as neuropsychological impairment, are related to its malfunctioning [53,54,55]. It is well known that schizophrenia is associated with abnormal structural and functional connectivity [56], although this can be partially restored by antipsychotic medication [45,57]. In this regard, nurses also play an important role in promoting medication adherence [58], and they may be among the first to detect non-adherence and non-attendance at follow-up visits [59]. Neurocognitive and social cognitive interventions also aim to improve cerebral functioning [60,61,62]. Psychological interventions produce changes in brain structure and its functioning [63,64], this being the goal of interventions such as cognitive remediation. The interventions have also been related to improvements in neurocognitive functioning [46], and could therefore influence these brain structures. Exercise also has important neurobiological effects [65]. The coordination of all these services and professionals by social workers will be crucial for improving cognitive outcomes [66]. Enhancement of this kind, in turn, leads to improved neurocognition and social cognition and a reduction in the negative symptoms [67,68,69], which is essential for functional recovery in individuals with schizophrenia. Therefore, from the expert perspective, the inclusion of this category (*s110 Structure of brain*) in the ICF-CS for schizophrenia should be considered. Nevertheless, we must bear in mind that many other categories that form part of the ICF-CS for schizophrenia do indirectly take this structure into account (for example, cognitive functions), given the correspondence between these functions and the underlying structures.

### 4.3. Activities and Participation

The *Activities and Participation* component is the one with the largest number of categories achieving expert consensus. In total, 39 categories achieved expert consensus, and of these, 31 achieved consensus among all professional panels considered, with seven of them achieving an average agreement higher than 95%.

The categories selected covered all the chapters of this component and focused especially on chapters *d6 Domestic life*, *d7 Interpersonal interactions and relationships*, and *d8 Major life areas*, such as education and employment. Experts also emphasized the possible problems in areas covered by the chapters *d1 Learning and applying knowledge*, *d2 General tasks and demands*, and *d5 Self-care*. All the categories of this component for which consensus was reached are included in the ICF-CS for schizophrenia. This reflects the fact that schizophrenia may have major implications for everyday functioning in all these areas [70] and illustrates why the main long-term therapeutic goals of all health interventions for individuals with schizophrenia should go beyond remission of specific symptoms and focus on improving social functioning [71].

There are nine categories from the ICF-CS for schizophrenia that did not achieve expert consensus. These categories mainly related to simple activities, such as *d860 Basic economic transactions*, whereas consensus was achieved for the equivalent more complex categories (e.g., *d865 Complex economic transactions).* These results offer a more positive view of the abilities of people with schizophrenia, since they suggest that their difficulties mainly depend on the complexity of the task at hand.

Managing to restore the psychosocial functioning of individuals with schizophrenia through their performance in key areas of everyday activities, social achievement and social competence is the cornerstone of functional recovery [72,73]. All the professions play a key role in achieving this goal. Psychiatric medication has shown a positive effect on the functioning and quality of life of individuals with schizophrenia [74]. Different approaches of psychological treatment, such as cognitive-behavioral therapy, social skills training, cognitive remediation and social cognition training, have also been shown to be effective in improving psychosocial functioning and participation in community activities [75]. Nursing interventions can also enhance social functioning, for example, through shared decision making, which has been suggested to improve social perception [76]. Social work also plays a central role in the identification of problems that affect one in carrying out daily activities and in the implementation of individualized and collaborative intervention plans guided by an individual’s lifestyle and preferences [77]. Several studies have shown that the intervention of OTs enhances social functioning (improving interpersonal communication, in particular), the realization of daily activities, and working life [78]. Physiotherapists can also improve one’s ability to perform several daily activities, and their intervention has also been related to better functional outcomes [17].

Multiple interventions are therefore needed to achieve functional recovery, requiring integrated care and interdisciplinary intervention [19,79]. Ultimately, being able to perform daily activities and to participate in community life is the main goal of all people, and it is therefore not surprising that this component represents a priority area of work and a treatment objective of all health interventions.

### 4.4. Environmental Factors

The component with the second-highest number of categories showing consensus was *Environmental factors*: 33 achieved expert consensus, and 21 achieved consensus from all professional panels considered.

The agreed categories covered five of the six chapters of this component, with the only chapter with no category achieving consensus being *e2 Natural environment and human-made changes to the environment.* The selected categories concerned in particular the chapters *e3 Support and relationships*, *e4 Attitudes*, and the accessibility of health services (chapter *e5 Services, systems and policies*). These results confirm that health professionals attribute particular importance to the impact of environmental factors on the functioning of a person with schizophrenia [80,81]. In fact, authors such as Fleischhacker et al. [7] point out the necessity of combining integrated care with active engagement on the part of people with schizophrenia, their families, and their communities, highlighting the relevance of paying attention to environmental circumstances. All these elements working together should result in better lives for all those affected.

Family interventions, such as psychoeducation for relatives or multifamily group therapy, have proven to be effective in improving psychosocial functioning and promoting the well-being of people with schizophrenia, as well as that of their relatives [82,83]. Social workers also try to intervene in families and in other contextual factors and in how these factors affect the individual with schizophrenia [84], and nursing interventions have also been shown to facilitate the achievement of adequate social and therapeutic support [11].

All the categories from this component that form part of the ICF-CS for schizophrenia achieved expert consensus. Only one category from this component that achieved expert consensus is not represented in the ICF-CS for schizophrenia (*e135 Products and technology for employment*).

All the areas selected may provide health professionals with information about how the individual functioning of persons with schizophrenia could be improved by promoting enabling environmental factors and by reducing barriers. In spite of the importance of these factors, they are usually ignored in the management of schizophrenia [85]. The ICF-CSs can be useful in this regard, since they enable a thorough assessment of the environmental barriers and facilitators that affect the daily functioning of a person with a specific health condition, identifying the environmental aspects which merit intervention.

### 4.5. Personal Factors

In total, 28 *Personal factors* reached expert consensus. Of these, 14 achieved an average consensus higher than 95% across the perspectives achieving consensus. This highlights the relevance of considering personal characteristics in the treatment of individuals with schizophrenia, as well as the importance attributed to this component by all the professional perspectives.

Many studies support the relevance of the *Personal factors* that achieved a higher consensus in our research, such as age at onset [86], premorbid drug use and lifestyle [87], premorbid social skills [88], premorbid cognitive skills [89] and personal history and biography [90,91]. Other categories that achieved high consensus, which are also supported by the literature, are resilience [92], genetic factors [93], premorbid personality [94] and premorbid intelligence [95].

Health professionals can, in fact, influence some of these personal factors. For example, psychosocial skills can be improved by psychologists (through social skills training or social cognition training [75], and nurses can also help to promote them through specific workshops [70]. Moreover, the knowledge of which personal factors may increase one’s vulnerability to having a psychotic episode is very important given that it will facilitate the identification of individuals who are at clinically high risk for schizophrenia as well as then the application of preventive approaches [96]. In such cases, social work holds a unique position when it comes to identifying these individuals and making appropriate referrals [97].

Consequently, it would be useful for the ICF system to incorporate the classification of this component, in order to enable the systematic identification of all personal factors that influence the functioning of people with different health conditions, so that professionals can describe them in a detailed and exhaustive manner. Most of the categories regarded as important in our study coincide among the different perspectives of the various health professions, which suggests that the proposed list of *Personal factors* captures the aspects that merit particular consideration in this population.

### 4.6. Strengths, Limitations and Future Research

The present study has several strengths. First, the Delphi technique was applied in such a way as to maximize its possibilities, by including a large number of participants and facilitating worldwide participation. To our knowledge, this is the first time that an ICF-CS validation study has been conducted on such a large and diverse sample. Moreover, the sample was made up of experts highly qualified in the treatment of individuals with schizophrenia, both acute and chronic, from both rural and urban settings. Another strength of the study is that participation was possible in five languages, and this is likely to have been a key factor in achieving such multicultural and multinational representation. It should also be noted that the response rate across rounds one to three was between 63.2% and 86.1%, which is considerably high for this kind of study. Finally, the proposed list of *Personal factors* constitutes a valuable and innovative aspect of our study.

Nonetheless, some consideration should be given to certain limitations of the study’s findings. The primary limitation concerns the representativeness of the panel of experts. Although individuals from all around the world took part, the African and Eastern Mediterranean WHO regions were under-represented. Possible reasons for this under-representation include greater difficulties in accessing these professionals and lower numbers of these kinds of specialized health professionals in these regions. Moreover, despite our efforts to recruit a large and representative panel of experienced professionals across all the areas, the number of physiotherapists and social workers willing to participate in the Delphi study was relatively small. The sample of occupational therapists and nurses was also small compared with the number of psychiatrists and psychologists. It is worth noting that mental health nurses make up the bulk of the mental health workforce, yet only 160 out of 1555 mental health professionals that initially agreed to participate were nurses. Although experts were recruited through many sources, including the scientific literature, international associations, and LinkedIn, in many of these sources, all these professionals remain underrepresented. This article attempts to be supportive by giving a voice to all these professional groups, in order to promote a multidisciplinary and integrative health care approach. 

In summary, these Delphi studies have documented the areas and aspects that health professionals consider important in relation to the assessment and treatment of individuals with schizophrenia, and the results largely validate the ICF-CSs for schizophrenia. Further validation studies from the perspective of families, caregivers and persons with schizophrenia, as well as studies that examine other sources of evidence of validity, are now needed in order to complement the present findings and to move towards a definitive version of the ICF-CS for schizophrenia.

## 5. Conclusions

Overall, the present study provides important support for the worldwide content validity of the ICF-CSs for schizophrenia from an expert perspective. The results highlight the relevance, in the evaluation and treatment of individuals with schizophrenia, of assessing functioning by considering the body functions, participation in activities, environmental aspects and personal factors that experts have identified. All this suggests that the ICF and these ICF-CSs provide an effective framework within which to evaluate and describe functioning in people with schizophrenia and therefore may be a useful tool in the comprehensive treatment of this population.

## Figures and Tables

**Table 1:** Socio-demographic and professional characteristics of the total sample of participants in the Delphi studies.

Professional Ambit	Round 1 *n*(%)	Women *n* (%)	Age Average (Rank)	Years of Experience Average (Rank)	WHO Region						Countries *n*	Treated Population ^g^				Round 3 *n* (%) ^h^
					African ^a^ *n* (%)	Americas ^b^ *n* (%)	Eastern Mediterranean ^c^ *n* (%)	European ^d^ *n* (%)	South-East Asia ^e^ *n* (%)	Western Pacific ^f^ *n* (%)		Acute *n* (%)	Chronic *n* (%)	Rural *n* (%)	Urban *n* (%)	
Psychiatry	352 (44.5)	99 (28.1)	47.6 (29–81)	19.5 (4–55)	26 (7.4)	72 (20.4)	17 (4.8)	82 (23.3)	77 (21.9)	78 (22.2)	63	325 (92.3)	315 (89.5)	207 (58.8)	303 (86.1)	303 (86.1)
Psychology	175 (22.2)	110 (62.9)	41.8 (24–67)	11.7 (1–42)	11 (6.3)	47 (26.9)	21 (12.0)	63 (36.0)	20 (11.4)	13 (7.4)	46	92 (52.6)	149 (85.1)	60 (34.3)	130 (74.3)	137 (78.3)
Nursing	101 (12.8)	64 (63.3)	45.8 (24–74)	20.7 (2–54)	5 (4.9)	25 (24.7)	9 (8.9)	31 (30.6)	13 (12.7)	18 (17.8)	30	82 (81.2)	89 (88.2)	45 (44.6)	69 (68.3)	79 (78.2)
Occupational therapy	92 (11.6)	76 (82.6)	37.7 (23–67)	9.9 (1–44)	13 (14.1)	16 (17.4)	7 (7.6)	42 (45.7)	5 (5.4)	9 (9.8)	29	49 (53.3)	79 (85.9)	31 (33.7)	60 (65.2)	73 (79.3)
Social work	57 (7.2)	39 (68.4)	45.1 (26–72)	10.3 (1–27)	2 (3.5)	17 (29.8)	1 (1.8)	13 (22.8)	11 (19.3)	13 (22.8)	20	28 (49.1)	53 (93.0)	24 (42.1)	43 (75.4)	36 (63.2)
Physiotherapy	13 (1.6)	7 (53.8)	43.2 (32–62)	10.5 (1–30)	0	2 (15.4)	0	7 (53.8)	1 (7.7)	3 (23.1)	8	7 (53.8)	12 (92.3)	3 (23.1)	7 (53.8)	10 (76.9)
Total	790	307 (48.1)	45.5 (23–81)	15.8 (1–55)	57 (7.2)	179 (22.6)	55 (7.0)	238 (30.1)	127 (16.1)	134 (17.0)	85	583 (73.8)	697 (88.2)	370 (46.8)	612 (77.5)	638 (80.8)

^a^ Participating countries in the African region: Algeria, Botswana, Ethiopia, Ghana, Kenya, Mozambique, Nigeria, South Africa, Uganda, Zimbabwe. ^b^ Participating countries in the Americas region: Argentina, Brazil, Canada, Chile, Colombia, Costa Rica, Cuba, Ecuador, Mexico, United States of America, Uruguay, Venezuela. ^c^ Participating countries in the Eastern Mediterranean region: Egypt, Iran, Iraq, Jordan, Kuwait, Lebanon, Libya, Marocco, Pakistan, Saudi Arabia, United Arab Emirates. ^d^ Participating countries in the European region: Armenia, Belgium, Bosnia and Herzegovina, Bulgaria, Croatia, Cyprus, Czechia, Denmark, Finland, France, Georgia, Germany, Greece, Hungary, Iceland, Ireland, Israel, Italy, Latvia, Lithuania, Macedonia, Netherlands, Norway, Poland, Portugal, Romania, Russian Federation, Serbia, Slovakia, Slovenia, Spain, Sweden, Switzerland, Turkey, Ukraine, United Kingdom. ^e^ Participating countries in the South-East Asia region: Bangladesh, India, Indonesia, Nepal, Sri Lanka, Thailand. ^f^ Participating countries in the Western Pacific region: Australia, Cambodia, China, Japan, Malaysia, New Zealand, Philippines, Singapore, Republic of Korea, Taiwan. ^g^ It was possible to choose more than one option. ^h^ Regarding round 1.

**Table 2:** Number of categories that reached expert consensus and comparison with the categories included in the complete version of the ICF-CS for schizophrenia.

Number of Categories	Body Functions	Body Structures	Activities and Participation	Environmental Factors	Total
Categories that achieved consensus from at least one professional perspective	21	2	50	40	113
Categories in the ICF-CS for schizophrenia	17	0	48	32	97
Categories that achieved expert consensus ^a^	17	1	39	33	90
ICF-CS categories for which expert consensus was achieved	16	0	39	32	87

^a^ Expert consensus: consensus by four or more expert perspectives regarding that category.

**Table 3:** Categories that did not match between the set of categories that achieved expert consensus and the ICF-CS for schizophrenia.

	ICF Component	ICF Category	Perspectives from Achieving Consensus	Number of Perspectives from Which Achieved Consensus
Categories that achieved expert consensus ^a^ and are not present in the ICF-CS	Body functions	b126 Temperament and personality functions	All	6
	Body structures	s110 Structure of brain	All	6
	Environmental factors	e135 Products and technology for employment	PC, PS, NS, OT	4
ICF-CS categories for schizophrenia that did not achieve expert consensus	Body functions	b530 Weight maintenance functions	PC, NS, PH	3
	Activities and Participation	d330 Speaking	SW, PH	2
		d475 Driving	OT, PH	2
		d510 Washing oneself	OT, SW	2
		d540 Dressing	NS, OT	2
		d166 Reading	PH	1
		d210 Undertaking a single task	OT	1
		d470 Using transportation	OT	1
		d860 Basic economic transactions	OT	1
		d930 Religion and spirituality	OT	1

^a^ Expert consensus: consensus by four or more expert perspectives regarding that category. PC: psychiatry; PS: psychology; NS: nurses; OT: occupational therapy; SW: social work; PH: physiotherapy.

## Data Availability

The data presented in this study are available on request from the corresponding author. The data are not publicly available due to containing information that could compromise the privacy of research participants.
